# A del(X)(p11) carrying *SRY *sequences in an infant with ambiguous genitalia

**DOI:** 10.1186/1471-2431-6-11

**Published:** 2006-04-04

**Authors:** M Ellaithi, D Gisselsson, T Nilsson, S Abd El-Fatah, T Ali, A Elagib, ME Ibrahim, I Fadl-Elmula

**Affiliations:** 1 Institute of Endemic Diseases, University of Khartoum, Khartoum, Sudan; 2 International University of Africa, Faculty of Medicine and Health Sciences, Khartoum, Sudan; 3 The Orchids society for congenitally malformed children, Khartoum, Sudan; 4 Department of Clinical Genetics, University Hospital, Lund, Sweden; 5 Khartoum Teaching Hospital, Department of Radiology, Khartoum, Sudan; 6 Tropical Medical Research Institute, Khartoum, Sudan; 7 Al Neelain Medical Research Center, Faculty of Medicine, Al Neelain University, Khartoum, Sudan

## Abstract

**Background:**

*SRY *(sex-determining region, Y) is the gene responsible of gonadal differentiation in the male and it is essential for the regular development of male genitalia. Translocations involving the human sex chromosomes are rarely reported, however here we are reporting a very rare translocation of SRY gene to the q -arm of a deleted X chromosome. This finding was confirmed by cytogenetic, fluorescent *in situ *hybridization (FISH) and polymerase chain reaction (PCR).

**Case presentation:**

A 7-month infant was clinically diagnosed as an intersex case, with a phallus, labia majora and minora, a blind vagina and a male urethra. Neither uterus nor testes was detected by Ultrasonography. G-banding of his chromosomes showed 46,X,del(X)(p11) and fluorescent *in situ *hybridization (FISH) analysis showed a very small piece from the Y chromosome translocated to the q-arm of the del(X). Polymerase chain reaction (PCR) analysis revealed the presence of material from the sex-determining region Y (SRY) gene.

**Conclusion:**

It is suggested that the phenotype of the patient was caused by activation of the deleted X chromosome with *SRY *translocation, which is responsible for gonadal differentiation.

## Background

The *SRY *(sex-determining region Y), which is normally located in the distal part of the short arm of the Y chromosome is a genetic 'master switch' of gonadal differentiation [1], the product of which is present in the male genital ridge before testis formation and is required for the regular development of male genitalia [2]. *SRY *encodes a transcription factor that is a member of the high mobility group (HMG)-box family of DNA binding proteins and in mammals triggers the development of undifferentiated gonads towards a testicular phenotype [1,3]. In humans, zygotes bearing mutations in SRY develop into XY females [4,5], while XX individuals with the presence of *SRY *typically show a normal male phenotype [6], but may occasionally show ambiguous genitalia [7]. One example is testicular regression syndrome (XY gonadal regression syndrome), in which there are no gonads, and variable development of Mullerian and Wolfian ducts depending on the stage of fetal development at which the embryonic testis involutes; in most cases this happens before Mullerian tissues have regressed and before testosterone synthesis has started, the etiology is unknown but affected siblings have been reported [8].

## Case presentation

### Case history

**Figure 1:**
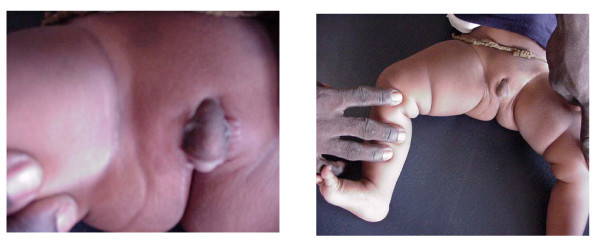
The external genitalia of the 7- month Sudanese infant who was clinically diagnosed as intersex.

A 7- month old infant, raised as a male, was clinically diagnosed as an intersex case. On examination the patient showed a big phallus, labia majora and minora, a blind vagina and a male urethra (Fig. 1). Ultrasonographic investigations did not detect the presence of uterus or testes. The patient was referred for chromosomal analysis.

#### Cytogenetic analysis

Patient peripheral blood was subjected to short-term culturing in RPMI 1640 medium for 72 hours. After metaphase arrest through exposure to Colcemide, cells were harvested, treated with hypotonic solution, and then fixed with methanol and acetic acid according to standard procedures. The harvested cells were dropped on clean slides and stained with Wright's stain, for chromosome banding [9]. The clonality criteria and the karyotypic descriptions were according to the ISCN recommendations [10].

#### Fluorescent in situ hybridization (FISH)

FISH with whole chromosome painting, and X centromeric probe, and the *SRY *gene specific probe (Vysis, Naperville, IL) was applied to fixated metaphases cells according to standard procedures [11].

#### Polymerase chain reaction (PCR)

DNA extraction and amplification methods were according to [12]. Primers were designed for PCR amplification using Genosys primer.3 software (Table 1).

**Figure 2:**
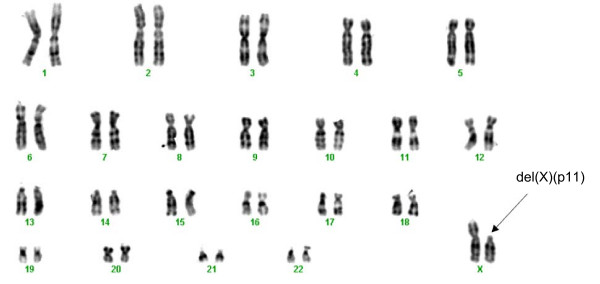
Metaphase cell showing 46,X,del(X)(p11).

Analysis of 11 metaphase cells showed 46,X,del(X)(p11) in all cells, which is a deletion within the breakpoint in band 11 in the p arm of one of the X chromosomes (Fig 2).

**Figure 3:**
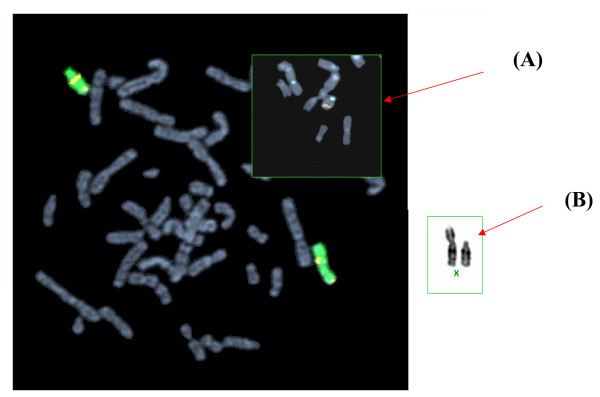
FISH analysis by whole chromosome painting shows a translocation of *SRY *material to the q-arm of the del(X). The X chromosome is painted in green and the Y chromosome in red/orange. Normal cross hybridization of the Y painting probe is seen in proximal Xq of both the normal X chromosome and the del(X), whereas normal cross hybridization to Xp is only seen in the normal X chromosome as these sequences are missing from del(X). The del(X) also shows a signal at distal Xq, corresponding to translocated Y sequences. Inset (A): *SRY *material (red/orange) is located at distal Xq while X centromere is in green. Inset (B): The G- banding of del(X) and normal X.

FISH analysis with whole chromosome painting probes and gene specific probes for the *SRY *gene showed a translocation of *SRY *material to the q-arm of the del(X) (Fig 3).

**Figure 4:**
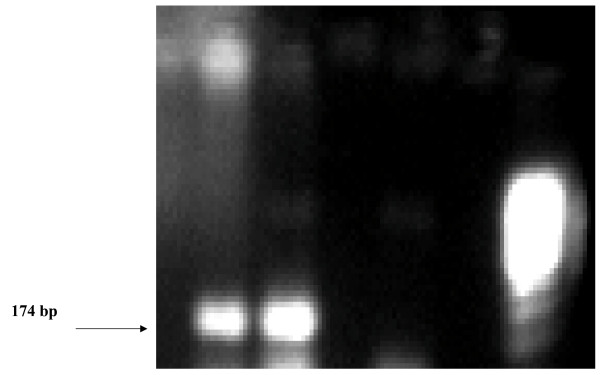
The PCR analysis showing the presence of *SRY *material in patient blood. *Lane 1 *patient sample, *lane 2 *positive control, *lane 3 *negative control, *lane *6 123 DNA markers.

PCR analysis corroborated the presence of *SRY *material in the peripheral blood of the patient (Fig 4).

## Conclusion

Translocations involving the human sex chromosomes are rarely reported. One of the best-known consequences of such exchanges is sex reversal in 46,XX males and some 46,XY females, due to exchange in the paternal germ line of terminal portions of Xp and Yp, including the SRY gene [13]. The presence of the *SRY *gene in the normal male zygote leads to the development of male genital organs and the absence of this gene leads to the development of the female genitalia. Patients who carry a structural abnormality of the X chromosome and ambiguous genitalia have provided opportunities to elucidate the genotype/phenotype correlation in relation to the X and Y chromosome content and X chromosome inactivation. At least one previous intersex case with an Xp deletion has been reported [14]. However, *SRY *sequences could not be detected in that patient and the authors suggested that the phenotype resulted from unmasking of recessive mutations in Xp by activation of the abnormal X chromosome in the majority of cells. In the present case, G-banding showed one ostensibly normal X chromosome and one X chromosome with deletion of most of the short arm. However, FISH and PCR analyses showed that there was also a translocation of *SRY *gene material to the long arm of abnormal X, which could potentially lead to development of normal male genitalia. In the present case, development of normal male genitalia presumes a skewed inactivation, allowing expression of the *SRY *in the gonadal cell population [15].

We hypothesize that the incomplete masculinisation of our patient is due to activation of the abnormal X to which *SRY *sequences had been translocated in a subpopulation of gonadal cells. This is the first case of this rare intersex condition reported from the Sudan.

## Competing interests

The author(s) declare that they have no competing interests.

## Authors' contributions

ME has collected the sample, cultured it, and harvested metaphase chromosomes; ME has also participated in the design and coordination of the study and drafted the manuscript. DG and TN performed the cytogenetic analysis; DG has performed FISH. DG and TN as also participated in the design of the study and helped to draft the manuscript. SA and ME has performed the PCR analysis, helped to draft the manuscript. TA has performed the ultarsonography and also helped to draft the manuscript. AE, and IF participated in the design of the study, and helped to draft the manuscript. All authors have given final approval of the final version of this paper.

## Note

***Table 1*: **Primer, DNA sequence, and temperature used in DNA analysis

## Pre-publication history

The pre-publication history for this paper can be accessed here:


